# Hepatitis B and C Viral Infection: Prevalence, Knowledge, Attitude, Practice, and Occupational Exposure among Healthcare Workers of Jimma University Medical Center, Southwest Ethiopia

**DOI:** 10.1155/2019/9482607

**Published:** 2019-02-03

**Authors:** Habtemu J. Hebo, Desta H. Gemeda, Kedir A. Abdusemed

**Affiliations:** ^1^Department of Epidemiology, Public Health Faculty, Institute of Health, Jimma University, P.O. Box 378, Jimma, Ethiopia; ^2^Department of Medical Laboratory Sciences and Pathology, Health Sciences Faculty, Institute of Health, Jimma University, P.O. Box 378, Jimma, Ethiopia

## Abstract

**Background:**

Blood-borne infections have been recognized as an occupational hazard for nearly 50 years. Current treatment for hepatitis B virus (HBV) is very expensive for individuals in developing countries and cannot clear infection after it progresses to the chronic stage. Thus, early screenings of people who are at higher risk like healthcare workers and vaccination and awareness creation on standard precautions (SP) to prevent transmission are mandatory. This study determined seroprevalence of HBV and hepatitis C virus (HCV) among healthcare workers of Jimma University Medical Center (JUMC).

**Methods:**

An institution based cross-sectional study was conducted from Nov 2015 to Jan 2016. The lottery method was used to select 240 healthcare workers. Data were collected by a self-administered questionnaire. Five to ten milliliters of whole venous blood was collected from each participant. The blood samples were analyzed (tested) for hepatitis B surface antigen (HBsAg) and anti-HCV antibody using automated Enzyme-Linked Immunosorbent Assay (ELISA). Data were entered into EpiData 3.1 and analyzed by SPSS 23.

**Results:**

The positivity of HBsAg was 2.5% (6/240; 95% CI: 0.52-4.48%) and that of anti-HCV antibody was 0.42% (1/240; 95% CI: 0.0-1.23%). Most participants had good knowledge of HBV (73.9%), HCV (60.9%), and SP (82.2%) and positive attitude towards SP (88.7%), but only 42.6% had a good practice of SP. More than half (60%) and nearly half (43%) had a history of ever exposure and exposure in the last one year before the survey, respectively. Females were at lower risk of both having ever exposure (95% CI: (0.241, 0.777)) and exposure in the last one year before the survey (95% CI: (0.297, 0.933)) compared to males.

**Conclusion:**

The prevalence of HBV was intermediate according to the endemicity classification by WHO. The practice of SP was poor in most participants and, thus, occupational exposure was high. Therefore, regular screening and vaccination of healthcare workers, regular provision of basic or refresher training and availing logistics, and regular motivation of healthcare workers on the practice of standard precautions are recommended.

## 1. Background 

In their occupational environment, healthcare workers (HCWs) are exposed to hazardous blood-borne pathogens such as hepatitis B virus (HBV) and hepatitis C virus (HCV). HBV and HCV are common causes of occupational diseases transmitted from patients to HCWs and vice versa and also to HCWs' families. HBV and HCV infections are serious public health problems that can have consequences in terms of psychological and occupational diseases [1]. HBV is contagious and can easily be transmitted from one infected individual to another by blood contact, from mother to child, by unprotected sexual intercourse, or by sharing of eating utensils and other barber shop and beauty salon equipment. The main transmission routes include prenatal infection, skin and mucous membrane infections caused by contaminated blood or body fluid, sexual contact, and injection drug abuse. In addition, tattooing, ear piercing, acupuncture, dialysis, and even use of a syringe can be the source of infection [2].

Hepatitis B is a very important public health problem affecting almost 10% of the world population. According to the 2009 WHO report, about 2 billion people are affected with HBV worldwide, more than 350 million suffered from chronic, lifelong infection, and more than one million individuals died because of cirrhosis and liver cancer every year [2–4]. It is also estimated that 170 million are chronically infected with HCV [5]. The burden of HBV infection is highest in the developing world, particularly in Asia and sub-Saharan Africa. WHO estimated that the prevalence of HBV infection in Africa is on average more than 10%. However, a study conducted in Addis Ababa, Ethiopia, showed that the mean prevalence of HBsAg was 6.1% [4].

The Centers for Disease Control (CDC) reported that 3.9 million individuals (1.8%) are infected with HCV, and 2.7 million of these infections will become chronic [1]. The prevalence of anti-HB virus antibody among volunteer blood donors ranged from 5 to 10%. But the prevalence is higher in persons from lower socioeconomic statuses, people of older age groups, and those persons exposed to blood products [2]. It has been estimated that 14.4% and 1.4% of hospital workers are infected with HBV and HCV, respectively [1]. Healthcare personnel, including support staff, who work in healthcare settings, represent a high-risk population for serious, potentially life-threatening infections such as HIV and HBV. Direct contact with blood and other body fluids is the most common or frequent risk healthcare workers encounter while caring for patients [3]. Studies in the United States have shown that the risk of acquiring HBV after being stuck with a needle from an HBV+ client ranged from 27 to 37%. In addition, the risk of acquiring HCV after being stuck with a needle from an infected person ranged from 3 to 10%. The efficiency for transmission of hepatitis B is high. For example, an accidental splash in the eye of as little as 10^−8^ ml of infected blood can transmit HBV to a susceptible host [6].

A safe and effective vaccine against HBV has been available for 20 years and is effective in preventing infection and the serious consequences of hepatitis, including liver cancer and cirrhosis, when given before or after exposure [2] but there is currently no vaccine for HCV [5]. Hepatitis B vaccine is recommended for pre- and/or postexposure prophylaxis of all persons at risk of contact with blood, blood products, or bodily secretions. Ideally, immunization against hepatitis B should be completed prior to health professional training because the risk of infection is thought to be higher at this time. Immunizing HCWs against hepatitis B prevents nosocomial transmission of the virus from HCWs to patients and from patients to HCWs as it gives long-term protection from hepatitis B infection, possibly lifelong [2, 7]. Since 1990 new hepatitis B infections among children and adolescents have dropped by more than 95% and by 75% in other age groups. However, in Ethiopia, the hepatitis B vaccine for children was introduced into the expanded program for immunization (EPI) program in 2007 [2]. There is not yet routine vaccination of HBV for all adults except for what is being done by some hospitals for their health professionals. There is not also yet postexposure prophylaxis (PEP) for HBV in Ethiopia. The current treatment for hepatitis B virus infection is very expensive for individuals in developing countries like Ethiopia and cannot clear infection after it progresses to a chronic stage [4]. Thus, early screenings of people who are at risk, such as healthcare workers, and the implementation of effective interventions such as vaccination and awareness creation for standard precautions to prevent transmission are mandatory.

Up to date, very few studies have been conducted to determine the seroprevalence of HBV and HCV in different population groups such as blood donors, medical waste handlers, VCT clients, commercial sex workers, and antenatal attendees in Ethiopia. Thus, the aim of this study was to determine the seroprevalence of HBV and HCV among healthcare workers in a specific study area. The study also aimed to assess the knowledge, attitude, practice, and occupational exposure of healthcare workers to guide interventions rendered at prevention and control of HBV and HCV infections in the health facilities.

## 2. Methods and Materials 

### 2.1. Study Area, Period, and Design

An institution based cross-sectional study was conducted from 11 Nov 2015 to 09 Jan 2016 at Jimma University Medical Center, 350 Kms southwest of Addis Ababa. There were a total of 810 healthcare workers (HCWs) (physicians, dentists, health officers, anesthetists, nurses, midwives, and laboratory personnel/technicians).

### 2.2. Population

#### 2.2.1. Source Population

The source population included all health workers (physicians, dentists, health officers, anesthetists, nurses, midwives, and laboratory technicians) at Jimma University Medical Center who had direct patient care or specimen contact during the study period.

#### 2.2.2. Study Population

The study population included all health workers as described under the source population and those who had direct patient care or specimen contact at least for the last 1 year.

### 2.3. Sample Size and Sampling Technique

#### 2.3.1. Sample Size

The sample size was calculated using a single population proportion formula assuming 8.7% prevalence (P) of HBV among healthcare workers [8], 5% level of significance (*α*), 3% margin of error (d), and 15% nonresponse rate.

(1) n=Zα/22P1−Pd2n=1.9620.0871−0.0870.032n=340.

When corrected using the finite population correction factor (because the size of the source population (N=606) was less than 10,000), the final sample size became 251.

#### 2.3.2. Sampling Technique

The sample size was proportionally allocated to each unit (wards, outpatient departments, and operation rooms). Then, the required number of study participants was selected by the lottery technique from each unit.

### 2.4. Variables

- Dependent variables
  - Seroprevalence of HBV
  - Seroprevalence of HCV
- Independent variables
  - Sociodemographic characteristics
    1. Age, sex, religion, ethnicity, marital status, profession, and work experience
  - History of occupational exposure
  - Knowledge of HBV and HCV transmission and prevention
  - Knowledge of standard precautions
  - Attitude towards standard precautions
  - Level of the practice of standard precautions

### 2.5. Measurements

Knowledge of hepatitis B and C virus was assessed by questions focusing on risk groups and procedures, transmission, treatment, and prevention of hepatitis B and C infection. There were 7 knowledge items for each virus and the total scores ranged from 0 (smallest) to 21 (largest). Knowledge, attitude, and practice of standard precautions were assessed by a set of questions and statements focusing on standard precautions. There were 8 knowledge items and the total scores ranged from 0 (smallest) to 27 (largest). There were 11 attitude statements whose responses were scored on the basis of a Likert scale. The scores ranged from 23 (strongly disagree) to 115 (strongly agree). There were 4 Likert scale questions to assess the frequency of compliance (practice) to standard precautions and the scores ranged from 0 (smallest) to 16 (largest).

Five to ten milliliters of whole venous blood was collected from each participant using a syringe, needle, and organ function test tube. The sample was transported to the JUMC Laboratory by a laboratory professional daily using a cold chain. Serum was separated from cells within an hour of arrival and serum samples were stored at -20 degrees Celsius. The HBsAg and anti-HCV-IgG antibody Enzyme-Linked Immunosorbent Assay (ELISA) was performed according to the manufacturer's instructions. Samples found positive at initial screening were analyzed again for the second time. Fortunately, all the samples found positive initially were found positive on repeated analysis.

### 2.6. Data Collection Plan

A self-administered structured English version questionnaire was used to collect data on sociodemographic characteristics, history of occupational exposure, knowledge of hepatitis B or C virus infections, and knowledge, attitude, and practice (KAP) of standard precautions. Data collection from HCWs was facilitated by two staff members (one healthcare worker who was not included in the study and a laboratory professional for blood collection) at each unit.

### 2.7. Data Analysis

Data were edited, entered into EpiData 3.1, exported to SPSS 20, and cleaned to check for completeness and extreme and missing values. Univariate analyses were performed and presented in tables, charts, and graphs. We could not conduct logistic regression because cases were limited (6 for HBV and 1 for HCV infection) for this purpose.

### 2.8. Data Quality Assurance

Before starting data collection, the research team thoroughly reviewed the questionnaire. One day orientation was given for facilitators and blood sample collectors to ensure a common understanding of the tool and methods of data collection. The questionnaire was pretested on 10% of the study participants who were not included in the actual study. The validity of the questionnaire was tested by an expert and peer review and the reliability (internal consistency) was checked by Cronbach's alpha coefficient. During the actual study, collected data were sorted and checked for errors and completeness on site daily. Overall data collection activities were supervised by the principal investigator. The participants anonymously responded to the items on the questionnaire. For laboratory data, specimen quality was checked for hemolysis, transportation conditions (temperature, tubes), and amount. HBsAg and anti-HCV-IgG antibody positive and negative controls were used for ELISA tests.

### 2.9. Ethical Considerations

The ethical clearance was secured from the College of Health Sciences Institutional Review Board (IRB), Jimma University. A letter of permission was submitted to the Jimma University Medical Center administration body and the administration bodies of each hospital's unit. The objective of the study was explained to each study participant. The participants were informed that there are interviews and blood sample collection for HBV and HCV screening. Written informed consent for participation was obtained from each study participant. The participation in the study was on a voluntary basis and the participants could withdraw from the study during the interview.

Participants were ensured that all collected data will be used only for the research purpose. Test results were kept confidential by using unique codes given to each study participant. Laboratory personnel had access only to the unique codes written on a sample containing test tubes. The research team members had access to both the unique codes and participants' identifiers (name and phone number) written in a separate format. All participants were informed of the result and those with a positive result were counseled and linked to care.

### 2.10. Plan for Data Dissemination

The finding of the study was reported to the Research Coordinating Office of Jimma University. A summary report was also submitted to Jimma University Medical Center.

### 2.11. Terms and Operational Definitions

- Good knowledge or good practice was scored as greater than or equal to 70%
- Positive attitude was scored as greater than 69 and negative attitude was scored as less than 69. A score equal to 69 was considered neutral
- Good practice was scored as greater than or equal to 60%

## 3. Result

### 3.1. Sociodemographic/Economic Characteristics

A total of 240 participants gave a sample and tested for both HBV and HCV, though 10 participants did not return the questionnaire. However, 11 (4.4%) healthcare workers refused to participate in the study. Most of the refusals (8) were from operation room healthcare workers and the remaining were from the ophthalmology, pediatrics, and psychiatry wards. The majority of participants (120 (52.2%)) were in the age group 25-29 years. The median age of participants was 25 (IQR=24-27) years. The proportion of males and females who participated in the study was almost the same. The leading proportion (102 (44.3%)) of participants was Orthodox Christian and most (137 (59.6%)) were Oromo. The majority of participants (134 (58.3%)) were single and the leading proportion (99 (43%)) was clinical nurses. The majority of participants (121 (52.6%)) had diploma level academic qualification and 148 (64.3%) had work experience of 1-3 years. The median work experience of participants was 3 years (IQR=2-5). A higher percentage (78 (33.9%)) had a monthly salary of 1601-3142 ETB and the mean (SD) of monthly salary was 2598.4 (1119.9) ETB (Table 1).

### 3.2. Risky Behavior and Medical Procedures

Most participants did not have risky behaviors and medical procedures, except for the injection of drugs where a considerable percentage (89 (38.7%)) of participants had it (Table 2).

### 3.3. Knowledge of Hepatitis B and C Viruses

#### 3.3.1. Knowledge of Hepatitis B Virus

The reliability (internal consistency) of knowledge items was good (Cronbach's alpha = 0.712). Most respondents (170 (73.9%)) had good overall knowledge about hepatitis B virus. However, only near to one-third (74 (32.2%)) of the respondents knew that it is not transmitted by contaminated water/food prepared by a person suffering from this infection. Similarly, only less than half (112 (48.7%)) of the respondents knew that HBV infection is treatable and only less than two-thirds (139 (60.5%)) knew that it has postexposure prophylaxis. The details of the responses to each question are indicated (Table 3).

#### 3.3.2. Knowledge of Hepatitis C Virus

The reliability (internal consistency) of knowledge items was good (Cronbach's alpha = 0.786). A majority of the respondents (140 (60.9%)) had good overall knowledge about hepatitis C virus. However, only a bit greater than one-fifth (52 (22.6%)) and less than one-fifth (34 (14.8%)) of the respondents knew that it is not transmitted by contaminated water/food prepared by a person suffering from this infection and breastfeeding, respectively. Similarly, only a bit greater than one-third (82 (35.7%)) knew that HBV infection is curable and only a bit greater than one-fourth (60 (26.1%)) knew that it has no postexposure prophylaxis. The details of the responses to each question are indicated (Table 4).

### 3.4. Knowledge, Attitude, and Practice of Standard Precautions

#### 3.4.1. Knowledge of Standard Precautions

The reliability (internal consistency) of knowledge items was good (Cronbach's alpha = 0.728). Most respondents (189 (82.2%)) had good overall knowledge about standard precautions. However, the level of knowledge in some components of standard precautions was not sufficient. For instance, only near to two-thirds (152 (66.1%)) of the respondents knew that standard precautions apply to blood, all body fluids, secretions, and excretions (except sweat), nonintact skin, or mucous membranes. Similarly, only less than half (103 (44.8%)) knew that standard precautions are intended not only for patients who have signs and symptoms of disease (s). Again, only a bit more than half (134 (58.3%)) of the respondents knew that needles should not be disposed mixed with other wastes/rubbish. The details of the responses to each question are indicated (Table 5).

#### 3.4.2. Attitude towards Standard Precautions

Attitude towards standard precautions was assessed by 23 items. The reliability (internal consistency) of items was good (Cronbach's alpha = 0.777). Respondents with a score greater than 69 (23*∗*3) were considered as having a positive attitude and those less than 69 were considered as having a negative attitude. Most of the respondents (88.7%) had a positive attitude towards standard precautions and only 8 (3.5%) were neutral. Positive attitude towards some components of standard precautions was not adequate. To mention just an example, only a bit greater than one-third (82, 35.7%) of the respondents disagree or strongly disagree that contaminated needles should be recapped immediately after use. The details of the responses to each statement are indicated (Table 6).

#### 3.4.3. Practice of Standard Precautions

Only less than half (98 (42.6%)) of the respondents had a good overall practice of standard precautions. Only less than half (94 (40.9%)) of the respondents usually or always practiced standard blood or body fluid precautions in their workplace. Similarly, only less than half (95 (41.3%)) of the respondents usually or always wore personal protective equipment (PPE) when needed. Details of the responses to each item are indicated (Table 7).

### 3.5. Occupational Exposure

More than half (60%) of the participants reported having ever exposure and 43% reported exposure in the last one year before the survey to blood or body fluid through splashing into the eyes and/or mouth or sharps or needlestick injury. A significant proportion (43%) of the respondents had a history of needlestick injury and the majority (56.6%) of this occurred within one year before the survey. More than one-third (38.3%) of the respondents had a history of sharp injury other than needlestick and the majority (57.95%) of this happened within one year before the survey. Nearly one-third (32.2%) of the respondents had a history of blood or body fluid splash into the eyes and/or mouth and nearly three-fourths (74.3%) of this occurred within one year before the survey (Table 8).

Among sociodemographic characteristics, only sex was associated with occupational exposure of the participants to blood or body fluid through splashing into the eyes and/or mouth or sharps or needlestick injury. Females were more than almost 57% lower at the risk of having ever exposure (AOR = 0.432; 95% CI: (0.241, 0.777)) and more than 42% lower at the risk of being exposed in the last one year before the survey (AOR = 0.527; 95% CI: (0.297, 0.933)) compared to males.

### 3.6. Prevalence of HBV and HCV Infection

Out of a total of 240 participants, 6 (2.5%; 95% CI: 0.52 to 4.48%) were positive for HBsAg whereas only 1 (0.42%; 95% CI: 0.0 to 1.23%) participant was positive for anti-HCV antibody. There was not any coinfection of HBV and HCV found among the participants. Five of those who were positive for HBsAg were in the age group 20-24 years and one was 48 years old. Five were males; 4 were single and 2 were currently married. Five of them were professional nurses and one was a psychiatry nurse. Five of them were BSc nurses and one was a diploma nurse. Five of them had work experience of 0-2 years and one had work experience of 32 years. All had an adequate overall knowledge of HBV and standard precautions whereas five had adequate knowledge of HCV. Five had a positive attitude towards standard precautions and one was neutral. Four of them had a good practice of standard precautions. Four had a history of ever needlestick injury; two had a history of sharp injury other than needlestick. Two had also a history of blood or body fluid splash into the eyes and/or mouth.

The only positive participant for HCV was a 25-year-old female. She had adequate knowledge of HBV, HCV, and standard precautions. She also had a positive attitude and good practice of standard precautions. She had no history of ever needlestick injury and did not remember the history of blood or body fluid splash into the eyes and/or mouth.

## 4. Discussion 

Studies conducted at different areas and on different populations regarding seroprevalence of hepatitis B and C viruses and their associated risk factors have reported different findings. Physicians, dentists, nurses, laboratory staff, and dialysis center personnel are at high risk of acquiring the infection. This study determined the prevalence of HBV and HCV infections among healthcare workers of a tertiary level public hospital.

The prevalence of HBV infection among healthcare workers was 2.5% indicating intermediate endemicity (2-7%) of the problem according to the WHO classification of the prevalence of HBV infection [9, 10] despite high (60%) prevalence of having ever been exposed to blood or body fluid through splashing into the eyes and/or mouth or sharps or needlestick injury. This could be because of lower prevalence of HBV infection in the general community and/or because most healthcare workers had a good overall knowledge of HBV and HCV and standard precautions and positive attitude towards standard precautions contrary to the report of FMOH, Ethiopia [6].

The prevalence of HBV infection determined in this study was comparable to the prevalence of 4.4% reported among healthcare workers in Khartoum, Sudan [11], and the prevalence of 2.9% reported among healthcare workers of a tertiary hospital in Rwanda [12] and the 4.2% prevalence reported among medicine and health science students of Wollo University, Northeast Ethiopia [13]. However, it was lower than the prevalence of 7.3% reported among healthcare workers of Bule Hora Woreda, Southern Ethiopia [14], the prevalence of 8.7% reported among healthcare workers of the Najran region, Southwestern Saudi Arabia [8], the prevalence of 8.1% reported among healthcare workers of a tertiary hospital in Uganda [15], and the prevalence of 7.0% reported among healthcare workers of a tertiary hospital in Tanzania [16]. On the other hand, it was higher than the 1.0% and 0.4% prevalence reported among healthcare workers of a tertiary care hospital in India [17, 18]. This could be because of differences in the level of knowledge of HBV and standard precautions and attitude and practice of standard precautions and occupational exposure.

The prevalence of HCV infection in this study (0.42%) was similar to the 0% prevalence report among healthcare workers of the Najran region, Southwestern Saudi Arabia [8], the prevalence of 1.3% among healthcare workers of a tertiary hospital in Rwanda [12], and the prevalence of 0.7% among medicine and health science students of Wollo University, Northeast Ethiopia [13]. This could be because students and healthcare workers might have a similar level of knowledge and practice of standard precautions and, thus, might have similar exposure and infection.

*Limitations*. This study might underestimate the true prevalence of both HBV and HCV infections as eight healthcare workers working in the operation room refused to participate in the study suspecting that they were already infected and did not want to know their status. The study might also have been underpowered for detecting cases of HCV as the sample size calculated for HBV was used because of resource scarcity. HBsAg positivity indicates either acute (active) or chronic infections. HBsAg negativity also indicates either the true absence of infection (susceptibility) or immunity due to vaccination or immunity due to resolved infection. These statuses can be differentiated by performing HBsAb, HBcAb, and HBcAb IgM tests. However, we did not perform these tests because of resource limitation. We might have also overestimated the practice of standard precautions as it was self-reported (social desirability bias) by the respondents and no attempt was made to directly observe actual practice. The same was true for the history of occupational exposure which was also self-reported (recall bias) by the respondents and no attempt was made to revise their health records.

## 5. Conclusion 

This study has determined the prevalence of HBV and HCV infections among healthcare workers and found intermediate endemicity of HBV infection and nonnegligible prevalence of HCV. The practice of standard precautions was also poor in most participants and, thus, occupational exposure was high. Thus, we recommended that JUMC regularly screen healthcare workers and avail vaccine which was started soon after completion of this study and interrupted later. We also recommend that JUMC and other interested stakeholders regularly provide basic or refresher training for healthcare workers on blood-borne infections and their effective prevention methods (standard precautions). JUMC should also avail logistics and regularly motivate healthcare workers on the practice of standard precautions.

## Figures and Tables

**Table 1:** Distribution of sociodemographic/economic characteristics of healthcare workers of JUMC, 11 Nov 2015 to 09 Jan 2016.

**Sociodemographic **	**Number**	**Percent**	**Sociodemographic **	**Number**	**Percent**
**characteristics**			**characteristics**		
**Age (years)**			**Profession **		
20-24	82	35.7	Clinical nurse	99	43.0
25-29	120	52.2	Midwifery nurse	30	13.0
30+	28	12.2	Professional nurse	77	33.5
**Sex **			Others	24	10.4
Male	116	50.4	**Academic qualification**		
Female	114	49.6	Diploma	121	52.6
**Religion **			BSc	101	43.9
Muslim	55	23.9	MSc/MPH	5	2.2
Orthodox	102	44.3	Specialist MD	3	1.3
Protestant	67	29.1	**Work experience (years)**		
Others (Catholic & Wakefeta)	6	2.6	1-3 years	148	64.3
**Ethnicity **			4-6 years	49	21.3
Oromo	137	59.6	6+ years	33	14.3
Amhara	53	23.0	**Monthly salary**		
Others	40	17.4	< 1301	21	9.1
**Marital status**			1301-1600	31	13.5
Single	134	58.3	1601-3142	78	33.9
Currently married	91	39.6	3143-3901	67	29.1
Separated/divorced	2	0.9	>3901	33	14.3
Widowed	3	1.3			

n =230 (denominator used to calculate the percentages), BSc = Bachelor of Science, MSc = Master of Science, MPH = Master of Public Health, and MD = Medical Doctor.

**Table 2:** Distribution of risky behaviors and medical procedures among healthcare workers of JUMC, 11 Nov 2015 to 09 Jan 2016.

**Risky behaviors and medical procedures**		Number	Percent
Alcohol drinking	Yes	27	11.7
Cigarette smoking	Yes	12	5.2
Khat chewing	Yes	30	13.0
Shisha smoking	Yes	6	2.6
Multiple sexual partner/s	Yes	14	6.1
Practicing tattoos	Yes	10	4.3
IV drug use	Yes	6	2.6
Blood transfusion	Yes	9	3.9
Injection of any drug	Yes	89	38.7
Any incision/surgery	Yes	26	11.3
Any dental procedure	Yes	54	23.5

**Table 3:** Knowledge of healthcare workers of JUMC about hepatitis B virus, 11 Nov 2015 to 09 Jan 2016.

**Items **		**Number**	**Percent**
(1) Who are vulnerable groups to Hepatitis B virus (HBV) infection?			
Healthcare workers	Yes	209	90.9
Commercial sex workers	Yes	157	68.3
IV drug users	Yes	108	47.0
Students on clinical practice	Yes	134	58.3
(2) Which of the following procedures may expose to the Hepatitis B virus (HBV) infection?			
Injections	Yes	209	90.9
Blood sampling	Yes	192	83.5
Incisions/surgery	Yes	195	84.8
Tattooing	Yes	148	64.3
(3) Hepatitis B virus (HBV) can be transmitted through:			
Contaminated blood transfusion	Yes	228	99.1
Unsafe sexual contact/practice	Yes	212	92.2
Contaminated water/food prepared by a person suffering from this infection?	No	74	32.2
Sharing needles or other equipment used for injecting illegal drugs	Yes	218	94.8
Accidental stuck with a used needle or other sharp instruments that has an infected person's blood on it	Yes	222	96.5
Blood or body fluid splashes onto an exposed surface (eyes, mouth, or cut in the skin)	Yes	221	96.1
Birth canal during childbirth (mother to newborn)	Yes	204	88.7
Breastfeeding	Yes	190	82.6
Sharing grooming items such as razors or toothbrushes	Yes	208	90.4
(4) Is HBV infection treatable?	Yes	112	48.7
(5) Is HBV infection curable?	No	154	67.0
(6) Can we prevent HBV transmission?	Yes	208	90.4
(7) Does HBV have post-exposure prophylaxis?	Yes	139	60.4

**Table 4:** Knowledge of healthcare workers of JUMC about hepatitis C virus, 11 Nov 2015 to 09 Jan 2016.

**Items **		Number	Percent
(1) Who are vulnerable groups to Hepatitis C virus (HCV) infection?			
Healthcare workers	Yes	174	75.7
Commercial sex workers	Yes	131	57.0
IV drug users	Yes	94	40.9
Students on clinical practice	Yes	116	50.4
(2) Which of the following procedures may expose to the Hepatitis C virus (HCV) infection?			
Injections	Yes	191	83.0
Blood sampling	Yes	177	77.0
Incisions/surgery	Yes	178	77.4
Tattooing	Yes	135	58.7
(3) Hepatitis C virus (HCV) can be transmitted through:			
Contaminated blood transfusion	Yes	210	91.3
Unsafe sexual contact/practice	Yes	196	85.2
Contaminated water/food prepared by a person suffering from this infection?	No	52	22.6
Sharing needles or other equipment used for injecting illegal drugs	Yes	200	87.0
Accidental stuck with a used needle or other sharp instruments that has an infected person's blood on it	Yes	208	90.4
Blood or body fluid splashes onto an exposed surface (eyes, mouth, or cut in the skin)	Yes	205	89.1
Birth canal during childbirth (mother to newborn)	Yes	187	81.3
Breastfeeding	No	34	14.8
Sharing grooming items such as razors or toothbrushes	Yes	192	83.5
(4) Is HCV infection treatable?	Yes	141	61.3
(5) Is HCV infection curable?	Yes	82	35.7
(6) Can we prevent HCV transmission?	Yes	207	90.0
(7) Does HCV have post-exposure prophylaxis?	No	60	26.1

**Table 5:** Knowledge of healthcare workers of JUMC about standard precautions, 11 Nov 2015 to 09 Jan 2016.

**Items **		Number	Percent
(1) **Standard precautions**:			
Are first level precautions designed for use in caring for all people – both clients and	Yes	166	72.2
patients attending healthcare facilities			
Apply to blood, all body fluids, secretions and excretions (except sweat), non-intact	Yes	152	66.1
skin or mucous membranes.			
Are second level precautions intended for use in patients known or highly suspected	No	72	31.3
of being infected or colonized with pathogens transmitted by air, droplet or contact			
Are intended only for patients who have signs and symptoms of disease (s)	No	103	44.8
(2) **Hand hygiene** (hand washing with soap and water or use of an antiseptic hand rub) is mandatory:			
After touching blood, body fluids, secretions, excretions, and contaminated items	Yes	205	89.1
Immediately after removing gloves	Yes	194	84.3
Between patient contacts	Yes	177	77.0
(3) **Gloves **are used:			
For contact with blood, body fluids, secretions/excretions or contaminated items	Yes	215	93.5
For contact with mucous membranes or non-intact skin	Yes	207	90.0
For any contact with patients	Yes	190	82.6
(4) **Gown/Apron** is used to:			
Protect skin from blood or body fluid contact	Yes	213	92.6
Prevent soiling of clothing during procedures that may involve contact with blood	Yes	207	90.0
or any body fluids (secretions/excretions)			
For any contact with patients	No	55	23.9
(5) **Mask, goggles** or **face shield** are used to protect	Yes	206	89.6
mucous membranes of eyes, nose or mouth when			
contact with blood or body fluids is likely			
(6) Which of the following is **Safe Injection Practices**?			
Avoid recapping, bending, breaking, or hand manipulating used needles	Yes	191	83.0
If recapping is required, use a one-handed scoop technique only	Yes	176	76.5
Avoid removing used needles from disposable syringes	Yes	184	80.0
Place used needles in a puncture-resistant container at the point of use	Yes	184	80.0
(7) Where/how should **needles be disposed**?			
Open pail	No	123	53.5
In sharp and liquid proof container without removing the syringe	Yes	165	71.7
In sharp and liquid proof container after separating the needle from the syringe	No	99	43.0
Mixed with other wastes/rubbish	No	134	58.3
(8) Which of the following is a **component of Standard Precautions** to prevent			
occupationally transmitted Blood Borne Infections?			
Hand hygiene	Yes	215	93.5
Using Gloves	Yes	223	97.0
Using Aprons	Yes	218	94.8
Safe disposal of sharp	Yes	219	95.2
Safe disposal of needles	Yes	218	94.8

**Table 6:** The attitude of healthcare workers of JUMC towards standard precautions, 11 Nov 2015 to 09 Jan 2016.

**Items **	Strongly disagree	Disagree	Neutral	Agree	Strongly agree
Since no one really knows what organism's clients or patients may have, it is essential that standard precautions be used all the time.	0	9	17	61	143

In the absence of compliance to standard precautions, healthcare facilities can be the source of infection and epidemic diseases	7	10	19	59	135

The glove should be used by all patient care contacts as a useful strategy for reducing the risk of transmission of organisms	1	6	15	55	153

Physical barriers (protective goggles, face masks or aprons) should be used if splashes or spills of any body fluids (secretions or excretions) are likely.	3	14	13	43	157

	Strongly agree	Agree	Neutral	Disagree	Strongly disagree

It is safe to use syringe between patients if the needle is changed	63	40	16	28	83

To prevent accidental injury, contaminated needles should be recapped immediately after use	95	32	21	31	51

**Hand washing**:					

Hand washing between every patient encounter is unnecessary	41	31	9	52	97

Hand washing does not affect clinical outcome	33	28	9	60	100

Hand washing is unnecessary when gloves are worn	27	30	16	61	96

Routine or frequent hand washing is unnecessary	29	22	13	66	100

Frequent hand washing interrupts efficient patient care	31	33	15	63	88

Frequent hand washing damages skin and causes cracking, dryness, irritation, and dermatitis	32	46	24	57	71

Hand washing damages nails and nail polish	21	25	18	75	91

Hand washing is inconvenient	35	37	28	67	63

Hand washing takes too much time	34	32	17	76	71

	Strongly disagree	Disagree	Neutral	Agree	Strongly agree

Wearing gloves do not replace the need for hand hygiene	30	48	20	60	72

A separate pair of gloves must be used for each client.	18	34	12	55	111

A separate pair of gloves must be used when moving from one site to another site on the same patient (i.e., from respiratory care to a dressing change)	32	49	17	47	85

**Sharps container should be:**					

Put as close to the point of use as possible, ideally within arm's reach.	21	22	15	79	93

Attached to walls or other surfaces if at all possible.	13	27	31	79	80

Marked clearly so that people will not unknowingly use them for discarding other items.	16	15	27	69	103

Marked the fill line at the three-quarters full level.	8	26	27	63	106

Not be shaken to settle its contents and make room for more sharps.	16	20	26	73	95

**Table 7:** The practice of standard precautions by healthcare workers of JUMC, 11 Nov 2015 to 09 Jan 2016.

Items	Never	Rarely	Sometimes	Usually	Always
How frequently do you practice standard blood or body fluid precautions at your workplace?	41	27	68	22	72

How frequently do you wash your hands before examining a patient?	33	26	101	16	54

How frequently do you wear PPE when needed?	56	16	63	28	67

How frequently do you remove all finger rings, watches and bracelets during surgical hand scrub?	78	14	40	14	84

**Table 8:** Occupational exposure of healthcare workers of JUMC, 11 Nov 2015 to 09 Jan 2016.

**Type of exposure**		**Number**	**Percent**
Have you ever had a needle stick injury?	Yes	99	43
	No	126	56.1
	Don't remember	5	2.2

Have you faced a needle stick injury within the last one year?	Yes	56	24.3
	No	169	73.5
	Don't remember	5	2.2

Have you ever had sharp injury other than needle stick?	Yes	88	38.3
	No	130	56.5
	Don't remember	12	5.2

Have you faced a sharp injury within the last one year?	Yes	51	22.2
	No	162	70.4
	Don't remember	17	7.4

Have you ever had blood or body fluid splash into your eye and/or mouth?	Yes	74	32.2
	No	150	65.2
	Don't remember	6	2.6

Have you had blood or body fluid splash into your eye and/or mouth within the last one year?	Yes	55	23.9
	No	161	70.0
	Don't remember	14	6.1

NB: those who did not remember their exposure were considered as not exposed.

## Data Availability

All relevant data are within the manuscript.

## Abbreviations

AOR: Adjusted odds ratio
CI: Confidence interval
ELISA: Enzyme-Linked Immunosorbent Assay
ETB: Ethiopian Birr
FMOH: Federal Ministry of Health
HBsAg: Hepatitis B surface antigen
HBV: Hepatitis B virus
HCWs: Healthcare workers
HCV: Hepatitis B virus
HIV: Human Immunodeficiency Virus
JUMC: Jimma University Medical Center
KAP: Knowledge, attitude, and practice
Kms: Kilometers
SP: Standard precautions
VCT: Voluntary counseling and testing.
